# Candidate approaches for predicting vitiligo recurrence: an effective model and biomarkers

**DOI:** 10.3389/fimmu.2025.1468665

**Published:** 2025-02-06

**Authors:** Binhao Liu, Jiacheng Shen, Jiayu Li, Bowen Tian, Bin Zhou, Jiachen Gui, Zhimin Li, Yue Zhang, Wenzhi Hu, Qiang Li

**Affiliations:** ^1^ The Air Force Clinical College, Anhui Medical University, Hefei, China; ^2^ Department of Anesthesiology, Second Affiliated Hospital of Anhui Medical University, Hefei, China; ^3^ Graduate School, Air Force Medical University, Xi’an, China; ^4^ Department of Dermatology, 960th Hospital of the People's Liberation Army of China (PLA), Jinan, China; ^5^ Graduate School, Hebei North University, Zhangjiakou, China; ^6^ Department of Dermatology, Air Force Medical Center, People's Liberation Army of China (PLA), Beijing, China; ^7^ Department of Burn and Plastic Surgery, Air Force Medical Center, People's Liberation Army of China (PLA), Beijing, China

**Keywords:** vitiligo, recurrence, activity, biomarker, interferon gamma, interleukin 6

## Abstract

**Background:**

Vitiligo is a challenging chronic condition with unpredictable disease course and high propensity for relapse post-treatment. Recent studies have reported the biomarkers for disease activity, severity, and therapeutic response of vitiligo, yet very few have investigated cytokines as predictive biomarkers for disease recurrence in vitiligo. This study aims to explore cytokines that serve as biomarkers for disease recurrence and extend research on factors related to the disease’s activity.

**Methods:**

92 patients and 40 healthy controls were recruited at the Air Force Medical Center from September 20, 2023, to November 30, 2023. Ultrasensitive multiplex cytokine array was used to measure plasma concentrations of cytokines, including IFN-γ, CXCL9, CXCL10, CXCL11, IL-6, and IL-15.

**Results:**

IFN-γ, CXCL9, CXCL10, CXCL11, IL-6, and IL-15 were expressed at higher levels in the circulation of patients with both segmental and non-segmental vitiligo compared to healthy controls (p < 0.001). There were no significant differences in these cytokine levels between the two types of vitiligo. CXCL9 was associated with the activity of vitiligo (p = 0.027). Correlation analysis showed a positive relationship between IFN-γ, CXCL9, CXCL10, CXCL11, IL-6, and IL-15 in the plasma of patients with recurrent vitiligo. The expression of IFN-γ, CXCL9, CXCL10, CXCL11, and IL-6 was significantly higher in recurrent vitiligo than in cases of persistent stable vitiligo (p = 0.001, p = 0.003, p < 0.001, p = 0.002, p = 0.026, respectively), with ROC analysis demonstrating their predictive capability for vitiligo recurrence, with AUC values of 0.806, 0.773, 0.896, 0.785, and 0.709, respectively. Multivariate logistic regression model showed IFN-γ is an independent predictor for vitiligo recurrence [OR (95%CI) =1.051 (1.012~1.116)], with a prediction accuracy of 90.5% (38/42) on the training dataset and 88.9% (16/18) on the testing dataset.

**Conclusion:**

Plasma IFN-γ, CXCL9, CXCL10, CXCL11 and IL-6 might be potential biomarkers for vitiligo recurrence, with CXCL9 also associated with disease activity. Additionally, multivariate logistic regression model demonstrated that IFN-γ is an independent predictor of vitiligo recurrence and the model could be a candidate approach for predicting vitiligo recurrence.

## 1 Introduction

Vitiligo is an acquired autoimmune disease characterized by a loss of melanocytes in the skin and mucous membrane, resulting in patchy depigmentation, affecting 0.5-2% population worldwide (1–3). To date, vitiligo is still a refractory disease because of the high recurrence rate after treatment cessation and obscure pathogenesis. Although, conventionally, it was considered that the pathogenic mechanisms between segmental and non-segmental vitiligo are different, according to the latest understanding of pathogenesis in vitiligo, the onset and recurrence of two type vitiligo are both closely related to inflammatory and immunity-related mechanisms, especially T-cell immunity (4–7). The defect melanocytes were directly destructed by CD8+Tcells (6). Studies have demonstrated that the recruitment of T cells is mediated by IFN-γ via IFN-γ-regulated chemokines, such as C-X-C motif chemokine ligand (CXCL) 9 and CXCL10 (8–10). IFN-γ mainly derived from CD4+Tcell and CD8+Tcell that immune polarized to type-1-like in vitiligo (11). Then, IFN-γ activates the Janus kinase (JAK) signaling pathway, promoting the expression of its regulated chemokines, including CXCL9, CXCL10 and CXCL11 (9, 12, 13). IFN-γ also increased the expression of CXCR3, the receptor of CXCL9, CXCL10 and CXCL11, on the CD8+Tcells and melanocytes (8, 9, 14). Interplaying between IFN-γ and IL-6 is required for the expression of IFN-γ-regulated genes, which is a key part of autoimmunity (15, 16). The upregulation of IL-6 is also reported in vitiligo patients (17). The chemokines orchestrate CD8+Tcells positing in epidermis adjacent to melanocytes and mediate the apoptosis of melanocytes through specifically combining with CXCR3 (10). Recent study showed that the fibroblasts are essential for the process of aggregating CD8+Tcell through the IFN-γ-CXCL9/CXCL10-CXCR3 axis (18). Besides, as reported, curative effect of cultured melanocyte transplantation was associated with the level of CXCL9 in the skin blister fluid, that is, the patients with high level of CXCL9 preferred occurring re-depigmentation after melanocyte transplantation (19). The result indicated that CXCL9 might induce recurrence of vitiligo. Currently, tissue resident memory T cell (TRM) is regarded as the chief cause of vitiligo recurrence and persistent depigmentation (7, 20). TRM cells are incapable of inducing melanocytes apoptosis directly and more likely to recruit T cells from the circulation for exerting the attack by secreting IFN-γ and IFN-γ-regulated chemokines: CXCL9 and CXCL10 (21). A model study reported that the survival of TRM cells in lesion skin depended on IL-15. The blockage of IL-15 receptor significantly decreased TRM survival and reversed vitiligo (22). To date, a number of clinical researches have reported that IFN-γ and its induced chemokines, CXCL9 and CXCL10, are related to disease activity, severity and prognosis in vitiligo (8, 19, 23, 24). However, very few studies have investigated the cytokines and chemokines that serve as predictive biomarkers for disease recurrence in vitiligo. Based on the existed research on the pathogenesis of vitiligo, it was hypothesized in present study that IFN-γ, IFN-γ-regulated chemokines, IL-6 and IL-15 are associated with disease activity and also predictive biomarkers for disease recurrence in vitiligo. In this study, we first established a multivariate regression model to predict the recurrence of vitiligo. This research also provided clinical evidence that IFN-γ is the crucial part for vitiligo recurrence.

## 2 Methods

### 2.1 Subjects and study design

Ninety-two patients, including 55 patients with non-segmental vitiligo and 37 patients with segmental vitiligo, and 40 matched health controls were enrolled at the Air Force Medical Center from September 20, 2023, to November 30, 2023. Patients with other autoimmune diseases, either within the 6 months prior to blood collection or during the follow-up period, were excluded. Prior to their enrollment in this study, patients had already undergone medical record establishment and therapeutic follow-up at this hospital. Patients were categorized into two groups based on their clinical status: active and stable vitiligo. Active vitiligo was defined by patient self-reports of spreading and/or new lesions within the past 6 months and a Vitiligo European Task Force (VETF) spreading score of +1 to +4. Stable vitiligo was identified in patients who exhibited no increase in lesion size or number within the same period and had a VETF spreading score of -4 to 0 (25). All patients were untreated or had received only topical treatment in the preceding 3 months by collecting samples (8). To investigate the relationship between the vitiligo recurrence and inflammatory factors, we conducted 6 months’ follow-ups with patients in stable stage after collecting blood samples and disease condition were assessed every month. Patients with stable vitiligo who experienced disease progression within six months (criteria for progression were the same as those for active vitiligo) were defined as having a recurrence, while those who did not were defined as persistent stable. During the follow-up period, patients were treated exclusively with topical therapies.

The study protocol was approved by ethics review board of the Air Force Medical Center (2022-50-YJ01). All patients and healthy controls signed a written informed consent.

### 2.2 Plasma analytes collection

Peripheral blood samples from 92 patients and 40 healthy controls were collected between September 20, 2023, and November 30, 2023. Peripheral blood (10 cc) was collected from vitiligo patients and healthy donors using heparinized tubes. The collected blood was centrifuged at 4°C and 3000g for 20 minutes. After centrifugation, the supernatant (plasma) was carefully extracted and subjected to two additional centrifugation steps to ensure purity. The plasma samples were aliquoted and stored at -80°C until analysis to prevent degradation due to repeated freeze-thaw cycles.

### 2.3 Plasma inflammatory factor assay by meso scale discovery

The Meso Scale Discovery (MSD) platform was employed for the quantitative analysis of inflammatory biomarkers. Experiments were conducted using the QuickPlex SQ120 instrument and the corresponding multiplex assay kit (Catalog #K15AME-1). All plasma samples were processed according to the manufacturer’s protocol. Samples and standards were incubated in antibody-precoated microplates, followed by the addition of a luminescent substrate. The electroluminescent signals were captured using an MSD reader, and data were analyzed using MSD Workbench software through the standard curve method to determine the concentrations of each biomarker. This method allows for precise multiplex detection, ensuring higher sensitivity and lower background noise compared to conventional ELISA. The detection range were from 1.7 to 17000 pg/ml for IFN-γ, from 0.33 to 1980 pg/ml for IL-6, from 0.82 to 3030 pg/ml for IL-15, from 0.11 to 1060 pg/ml for CXCL9, from 0.49 to 6000 pg/ml for CXCL10 and from 1.5 to 5080 pg/ml for CXCL11.

### 2.4 Statistical analysis

Continuous variables were described using mean (SD) or median (interquartile range). Discrete variables were presented as counts (percentages). The Chi-square test was employed to compare categorical variables. Wilcoxon test was utilized to compare differences between two independent samples with non-normally continuous distribution. For more than two independent samples, the Kruskal-Wallis H test was applied. Dunn’s multiple comparison test was performed to conduct pairwise comparisons among three independent samples. To control for Type I error, *P* was adjusted using the Bonferroni correction. Pearson correlation analysis was performed to explore the linear relationship between two continuous variables. Receiver Operating Characteristic (ROC) curve analysis was utilized to determine the sensitivity and specificity of detection factors in assessing the recurrence of vitiligo patients. A binary logistic regression model was established, with significant detection factors in recurrent patients as independent variables and vitiligo recurrence as the dependent variable, to explore central predictors for vitiligo recurrence and to predict the probability of recurrence in the near future.

All statistical analyses were conducted using R version 4.3.2 and GraphPad Prism software. p < 0.05 was considered statistically significant.

## 3 Results

### 3.1 Baseline clinical characteristics of patients

The baseline clinical characteristics of the patients are presented in **Table 1**. Statistical analysis indicated no significant differences in age and gender between the groups. We also excluded the influence of disease duration, family history of vitiligo, and history of other autoimmune diseases. Further, no significant difference was found in the proportions of patients with different type vitiligo among the groups.

**Table 1:** Demographics of active vitiligo group, stable vitiligo group and controls group.

	Active vitiligo	Stable vitiligo	Health control	p value
Age (years), median (IQR)	25 (12-42)	23 (10-35)	23 (17-30)	p>0.05
Gender (Male/Female)	14/18	29/31	20/20	p>0.05
Type of vitiligo (segmental/non-segmental vitiligo)	13/19	24/36	–	p>0.05
Disease duration (years), median (IQR)	2.2 (1.08-4.98)	2.25 (1.20-4.03)	–	p>0.05
Family history of vitiligo	3/29	6/54	–	p>0.05
History of other autoimmune disease	8/24	14/46	–	p>0.05

### 3.2 Elevated expression of cytokines in plasma in patients with vitiligo

**Figure 1:**
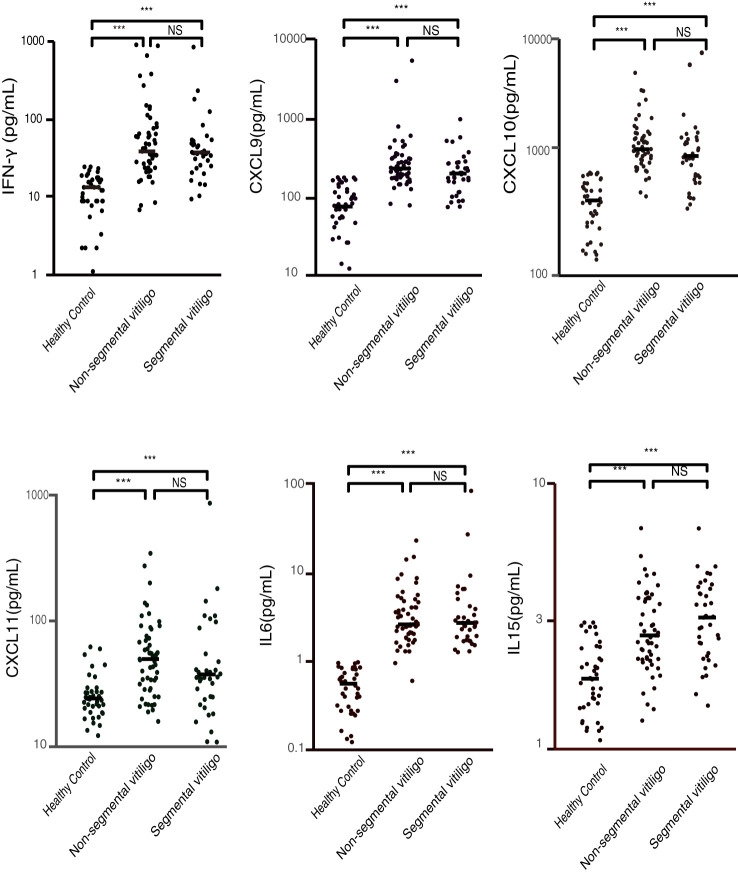
Plasma concentrations of IFN-γ, CXCL9, CXCL10, CXCL11, IL-6, and IL-15 in patients with segmental vitiligo, non-segmental vitiligo, and healthy controls (pg/ml). The plasma levels of these cytokines and chemokines are significantly higher in both segmental and non-segmental vitiligo patients compared to healthy controls, with no significant differences between the segmental and non-segmental groups. (***p < 0.001; NS, non-significant).

To ascertain whether the selected cytokine levels differ between segmental and non-segmental vitiligo patients, we compared the levels of these cytokines in various groups (The statistical data are shown in **Supplementary Table 1**). We found that the expression of plasma IFN-γ, CXCL9, CXCL10, CXCL11, IL-6 and IL-15 was significantly elevated in patients with both segmental vitiligo and non-segmental vitiligo compared with healthy controls (**Figure 1**). The result also found that there was no significant difference in these concentrations between patients with segmental vitiligo and patients with non-segmental. To investigate whether the duration of disease affects cytokine levels in vitiligo patients, we conducted a correlation analysis between each detected cytokine and disease duration. The results showed that none of the cytokine levels demonstrated a significant correlation with the duration of disease (shown in **Supplementary Figure 1**).

### 3.3 CXCL9 is associated with activity of vitiligo

**Figure 2:**
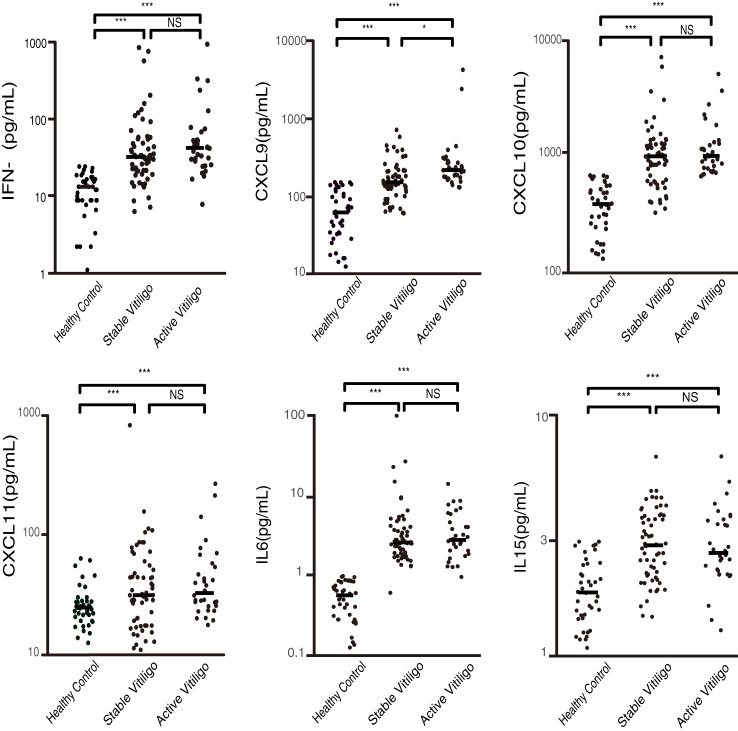
Plasma concentrations of IFN-γ, CXCL9, CXCL10, CXCL11, IL-6, and IL-15 in patients with active and stable vitiligo, and healthy controls (pg/ml). CXCL9 is associated with the activity of the disease, with significantly higher levels in patients with active vitiligo compared to those in stable and healthy controls. (*p < 0.05, ***p < 0.001; NS, non-significant).

Further, to verify whether these cytokines are associated with activity of vitiligo, we compared these cytokine levels between patients with active vitiligo, stable vitiligo and healthy controls (The statistical data are shown in **Supplementary Table 2**). All cytokine levels were significantly higher in patients with active and stable vitiligo compared to healthy controls (p < 0.001). Only the CXCL9 level was related to activity of vitiligo. The CXCL9 level in patients with active vitiligo was significantly higher than patients with stable vitiligo (p = 0.027) (**Figure 2**).

### 3.4 Correlation analysis of suspected cytokines in vitiligo recurrence

**Figure 3:**
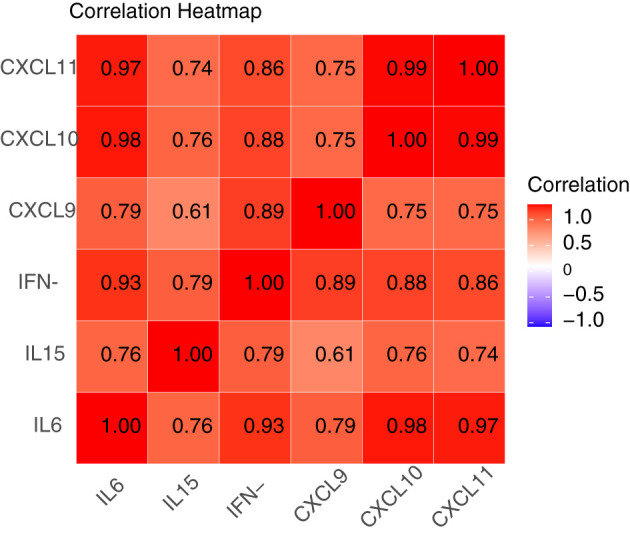
Correlation heatmap of IFN-γ, CXCL9, CXCL10, CXCL11, IL-6, and IL-15 in patients with recurrent vitiligo. A correlation coefficient between 0.5 and 0.7 indicates moderate correlation, while coefficients of 0.7 or higher indicate strong correlation. Apart from a moderate correlation between IL-15 and CXCL9 (correlation coefficient of 0.61), there exist strong correlations among IFN-γ, CXCL9, CXCL10, CXCL11, IL-6, and IL-15 (correlation coefficients >0.7).

As mentioned above, we have known that CXCL9 was related to vitiligo activity. Subsequently, we suspected whether increased levels of these immune factors would induce recurrence in patients with stable vitiligo. Based on previous research, we propose a hypothesis that IFN-γ, CXCL9, CXCL10, CXCL11, IL-6, and IL-15 are involved in the recurrence of vitiligo and are interconnected. Vitiligo recurrence was assessed through 6 months’ follow-up with patients in stable stage. To prove the proposed hypothesis, we conducted a correlation analysis of suspected factor levels in the plasma of recurrent patients. The results indicate positive correlations among these factors (**Figure 3**). Apart from a moderate correlation between IL-15 and CXCL9 (correlation coefficient of 0.61), strong correlations exist among the other factors (correlation coefficients >0.7).

### 3.5 Potential predictive biomarkers of recurrence in stable vitiligo

**Figure 4:**
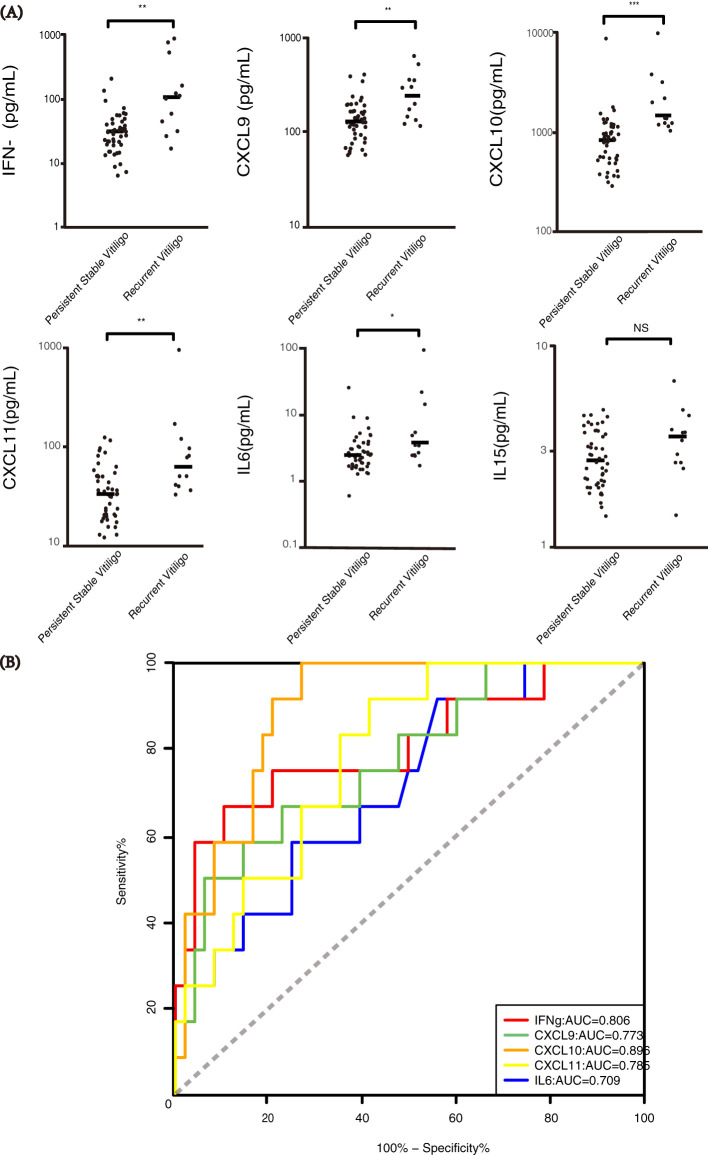
Association of IFN-γ, CXCL9, CXCL10, CXCL11 and IL-6 with recurrence in stable vitiligo patients. The number of recurrent patients and persistent stable patients were 12 and 48, respectively (12/48). **(A)** Plasma concentrations of IFN-γ, CXCL9, CXCL10, CXCL11, IL-6 and IL-15 in recurrent vitiligo and persistent stable vitiligo. The levels of IFN-γ, CXCL9, CXCL10, CXCL11 and IL-6 are significantly elevated in recurrent vitiligo. (*p < 0.05, **p < 0.01, ***p < 0.001; NS, non-significant). **(B)** ROC curve for disease recurrence in IFN-γ, CXCL9, CXCL10, CXCL11 and IL-6, insignificant data for IL-15 are not shown. The AUC (Area Under the Curve) values for these biomarkers are 0.806, 0.773, 0.896, 0.785, and 0.709 respectively.

We further compared the plasma concentrations of these factors in recurrent patients to concentrations in persistent stable patients. The plasma concentrations of IFN-γ, CXCL9, CXCL10, CXCL11 and IL-6 in recurrent patients were substantially elevated compared to persistent stable patients (p = 0.001, p = 0.003, p < 0.001, p = 0.002, p = 0.026, respectively), whereas there was no significant difference in circulating level of IL-15 (**Figure 4A**). Then, ROC curve analysis for IFN-γ, CXCL9, CXCL10, CXCL11 and IL-6 in recurrent versus persistent stable vitiligo were conducted. In the model with one predictor, CXCL10 is the most effective factor to predict the recurrence of vitiligo, with AUC value of 0.896. Additionally, IFN-γ, CXCL9, CXCL11, and IL-6 also exhibit substantial individual predictive capabilities for vitiligo recurrence, with AUC values of 0.806, 0.773, 0.785, and 0.709, respectively (**Figure 4B**).

### 3.6 Predictive multivariate logistic regression for vitiligo recurrence

**Figure 5:**
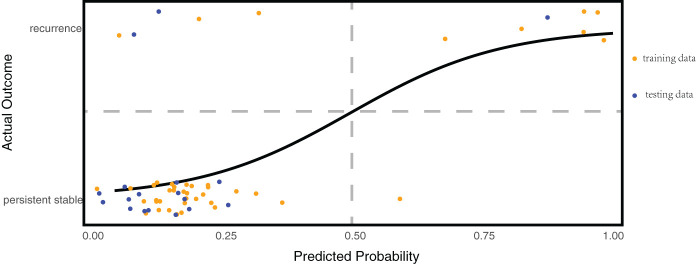
Multivariate regression predictive model for vitiligo recurrence. The horizontal axis represents the predicted probability calculated by the model, while the vertical axis shows the actual disease status of the patients. Patients are considered at risk of recurrence when the model’s predicted probability is ≥50%; they are considered persistent stable when the probability is <50%. This model achieved a prediction accuracy of 90.5% (38/42) on the training dataset and 88.9% (16/18) on the testing dataset.

To predict recurrence in patients with stable vitiligo more credible and eliminate the interrelations between predictors, we conducted multivariate logistic regression analysis. We randomly selected 70% of the stable patient cohort to serve as the training dataset for model development. The remaining 30% of the stable patients were used as the testing dataset to assess the trained model. The model’s performance was evaluated based on accuracy rate [(True Positive + True negative)/(True Positive + False Positive + True negative + False negative)]. The regression model revealed that IFN-γ was an independent predictor of vitiligo recurrence [OR (95%CI) =1.051 (1.012~1.116)], providing evidence that IFN-γ is the central trigger for vitiligo recurrence. The detailed results are shown in **Table 2**. To verify the model’s predictive performance, the model was utilized to calculate the probability of recurrence in patients with stable vitiligo. The formula for calculating the probability is given by probability = $\frac{1}{1+e^{-\beta }}$ , where $\beta =\beta 0+\beta_{\text{IFN}-\gamma }{\text{X}}_{\text{IFN}-\gamma }+\beta_{CXCL9}{\text{X}}_{CXCL9}+\beta_{CXCL10}{\text{X}}_{\text{CXCL}10}+\beta_{CXCL11}{\text{X}}_{CXCL11}+\beta_{IL-6}{\text{X}}_{IL-6}$ . The patients with high probability (predictive value ≥ 0.5) were considered as recurrent cases and with low probability (predictive value < 0.5) were considered as persistent stable cases. The results revealed that the trained model achieved a prediction accuracy of 90.5% (38/42) on the training dataset and 88.9% (16/18) on the testing dataset (**Figure 5**).

**Table 2:** Logistic regression model estimate for the probability of recurrence in patients with stable vitiligo.

Variable	Estimate	S.E	Z	P	OR (95%CI)
(Intercept)	-1.451	1.955	-0.742	0.458	0.234 (0.005~5.285)
CXCL11	0.017	0.021	0.842	0.410	1.017 (0.979~1.063)
IFNγ	0.051	0.025	2.028	0.043*	1.051 (1.012~1.116)
IL-6	-0.589	0.664	-0.888	0.375	0.555 (0.183~1.165)
CXCL10	-0.001	-0.001	-0.972	0.331	1.000 (0.999~1.001)
CXCL9	-0.011	0.011	-1.060	0.289	0.989 (0.963~1.007)

## 4 Discussion

Determining the status of vitiligo is fundamental for treatment. In current practice, disease stage is mainly assessed by clinical symptom based on patients’ self-reporting (26). However, the clinical manifestations of vitiligo lag behind melanocyte apoptosis (27), making it challenging to detect the progression of vitiligo early and prevent timely. Hence, we utilized the Meso Scale Discovery (MSD) platform to measure plasma concentrations of some vitiligo-associated cytokines, aiming to identify potential biomarkers that could assess the condition of vitiligo more sensitively and objectively.

The circulating concentrations of IFN-γ and its regulated chemokines are increased and correlate with disease activity in a number of autoimmune diseases, such as alopecia areata, systemic lupus erythematosus, and Graves’ disease (28–33). However, due to the detection limit with conventional ELISA, there exist few researches on IFN-γ as a biomarker (24, 34). A cytokine analysis study by Ng et al., using the MSD detection technology, found that IFN-γ is a sensitive and specific cytokine for reflecting vitiligo activity (24). MSD, a technology with low detection limits that are more suitable for samples with low endogenous levels of cytokines, were also employed in our study for multi-cytokine detection (35, 36). However, our study did not find a significant difference in plasma IFN-γ levels between patients with active and stable vitiligo, though the level of plasma IFN-γ was increased in vitiligo patients, which possibly due to differences in the definitions of active and stable vitiligo, their active vitiligo definition: patients experienced progression within 3 months; our active vitiligo definition: patients experienced progression within 6 months. In agreement with the previous studies, our results also showed that the plasma levels of CXCL9 and CXCL10 are increased in vitiligo patients (8, 19, 37). However, in contrast to the previous reports that patients with active vitiligo had higher expression of CXCL9 and CXCL10 compared to those with stable vitiligo (8, 38, 39), we only found CXCL9 expression is higher in the circulation of patients with active vitiligo compared to those with stable vitiligo, which may be related to the participants in our study, including segmental and non-segmental vitiligo patients. But a clinical study that included both segmental and non-segmental vitiligo patients, similar to our study, also showed that only serum CXCL9 levels correlated with vitiligo activity (19). The relationship between the expression of CXCL11 and vitiligo remains unclear because of the limited research on CXCL11 and vitiligo (40). But given its role in other autoimmune diseases and the relationship with CXCL9 and CXCL10, we also included CXCL11 in our study as a potential pathogenic factor (28, 41, 42). Rashighi et al. found that the expression of the CXCL11 gene is upregulated in lesional skin of vitiligo patients (28, 41, 42). Similarly, our results showed that the level of CXCL11 in plasma was elevated in vitiligo patients but no association with disease activity. Besides, almost consistent with previous results, our results also showed that the expression levels of IL-6 and IL-15 were significantly higher in vitiligo patients compared to healthy controls, with no significant difference between active and stable vitiligo (39); But a clinical study reported that IL-6 expression was significantly higher in active vitiligo compared to stable vitiligo (43). To exclude the potential impact of disease duration on cytokine levels, we performed further statistical analyses to examine the relationship between disease duration and cytokine levels. Our analysis did not reveal any significant correlation between cytokine levels and disease duration. However, Mascarenhas et al. reported a significant negative correlation between CXCL10 levels and disease duration (44). This discrepancy may be attributed to differences in the patient cohorts. In the study by Mascarenhas et al., the disease duration of recruited patients was evenly distributed over a range of 0–40 years, with most patients having a disease duration exceeding 10 years; In contrast, the majority of patients in our cohort had a disease duration of less than 10 years. Such a relatively short disease duration might not fully reflect the potential relationship between disease duration and cytokine levels.

In recent years, a central role of IFN-γ in vitiligo has been established (7). IFN-γ upregulates the expression of chemokines CXCL9 and CXCL10 through the JAK/STAT pathway to orchestrate CD8+ T cells positing in epidermis adjacent to melanocytes and facilitate their cytotoxic activity against surrounding melanocytes (10, 45). The interaction between IL-6 and IFN-γ is required for the expression of IFN-γ-regulated genes in autoimmunity (15). Besides, in the treatment of melanoma, immune checkpoint inhibitors often induce vitiligo-like skin lesions and patients with these lesions survive longer than those without (46, 47). Garris et al. reported that during programmed cell death protein 1 (PD-1) antibody treatment, the secretion of IFN-γ by CD8+ T cells is crucial for activating anti-tumor T cells, and neutralizing IFN-γ in mice impairs control over melanoma progression (48). Analysis of tumor microenvironment gene expression profiles revealed that tumors with higher levels of IFN-γ mRNA respond more effectively to PD-1 antibody therapy (49, 50). These results indicate that IFN-γ signaling is essential for the autoimmune-mediated destruction of melanocytes. Remarkably, tissue resident memory T cell (TRM) and recirculating memory T cells (TCM) also express IFN-γ, IFN-γ-induced chemokines and CXCR3 (20, 21, 51, 52), suggesting that IFN-γ and its inducible proteins may be involved in the maintenance and recurrence of vitiligo. Additionally, TRM cells express the CD122 subunit of IL-15 receptor. Richmond et al. reported that short-term blocking of IL-15 using anti-CD122 inhibits TRM production of IFN-γ and reverses vitiligo. Long-term blockade of IL-15 depletes TRM cells from skin lesions (22), indicating that IL-15 is essential for the survival of TRM cells. A mice model study demonstrated that JAK inhibitors reversed vitiligo but TRM cells are not depleted with the treatment (53). Another model study showed that blocking the recruitment or depleting TCM cells leads to repigmentation of vitiligo lesions, though the number of TRM cells in the lesions remains unchanged (21). Therefore, we propose a potential recurrence hypothesis: The binding of IL-15 to its receptor on TRM sustains survival of TRM cells and promotes the secretion of IFN-γ by these cells. The interaction between IFN-γ and IL-6 upregulates the expression of CXCL9, CXCL10, and CXCL11 in TRM cells and fibroblasts at the lesion site. These chemokines recruit TCM cells from lymph tissues to the vicinity of epidermal melanocytes by forming a gradient. Then, the recruited TCM cells attack the melanocytes and secrete IFN-γ, reinforcing the positive feedback loop.

If the hypothesis is confirmed, there should be correlations among the cytokines and chemokines involved. To test this hypothesis, we performed a correlation analysis of the plasma suspects expression in the recurrent patients. Consistent with expectations, the results showed strong positive correlations among these factors, providing indirect evidence for our hypothesis. Interestingly, IL-6 showed a strong positive correlation with the other five factors, which suggest that the interaction between IL-6 and IFN-γ in the induction of IFN-γ-regulated genes may also play a role in the pathogenesis of vitiligo recurrence. To further explore whether these suspected factors are triggers for vitiligo recurrence, we compared the plasma concentrations of these factors between recurrent and persistent stable patients. Our results showed that, except for IL-15, the levels of these factors were significantly higher in recurrent patients compared to persistent stable patients, which suggest that increased levels of IFN-γ, CXCL9, CXCL10, CXCL11 and IL-6 are potential triggers for vitiligo recurrence. ROC curve indicates that these five factors can serve as predictive biomarkers for vitiligo recurrence, providing an objective reference for assessing the disease status of vitiligo patients. Although IL-15 levels in recurrent patients showed an upward trend, the difference was not statistically significant. This may be due to the sustained high expression of IL-15 in stable patients to maintain TRM cell survival in lesional skin (22). In the process of recurrence, IL-15 may primarily act in the lesional skin, resulting in only a slight increase in circulation.

To predict vitiligo recurrence more reliably. we established a multivariate binary logistic regression model using the cytokines and chemokines significantly elevated in recurrent patients as independent variables and disease recurrence as the dependent variable. This model also eliminate collinearity among factors, identifying that IFN-γ is an independent predictor for vitiligo recurrence. Remarkably, the model demonstrated excellent predictive capability, with an accuracy of 90.5% on the training dataset and 88.9% on the testing dataset, which suggest that it is possible to predict the recurrence of vitiligo by monitoring the concentrations of cytokines and chemokines in the circulation, enabling timely intervention to prevent disease progression. But it should be noted that the sample size in this study is insufficient for the construction of the model, which may limit the model’s stability and generalizability, especially the potential issue of overfitting. However, our model adheres strictly to the established principles of predictive model and can provide a foundation for future larger-scale research.

There are several limitations in this study. Firstly, this is a single-center study, which may introduce a potential selection bias in the patient cohort. Secondly, the cohort size was relatively small, particularly for training the model, even though the model is theoretically supported. Moreover, our study only detected a few cytokines and chemokines closely related to vitiligo but did not screen all the cytokines that may play a role in pathogenesis of vitiligo. Above all, future large-scale studies based on multicenter and longitudinal samples are needed to investigate the plasma cytokines associated with vitiligo recurrence and validate the feasibility of multivariate model for predicting vitiligo recurrence.

In conclusion, this study discovers that plasma IFN-γ, IFN-γ-regulated chemokines, including CXCL9, CXCL10 and CXCL11, and IL-6 might be potential biomarkers for vitiligo recurrence, with CXCL9 also associated with disease activity. Additionally, our study also demonstrated that IFN-γ is an independent predictor of vitiligo recurrence and multivariate model could be a candidate approach for predicting vitiligo recurrence.

## Data Availability

The raw data supporting the conclusions of this article will be made available by the authors, without undue reservation.
