# Supplementary material for: Candidate approaches for predicting vitiligo recurrence: an effective model and biomarkers
Source: Front Immunol. 2025 Feb 6;16:1468665. doi: 10.3389/fimmu.2025.1468665 (PMC11839629; doi:10.3389/fimmu.2025.1468665)
Supplement: Supplementary file 1 [file Presentation1.zip › FIG1 statistical data.docx]

|  | Non-segmental Vitiligo (pg/ml) | Segmental Vitiligo (pg/ml) | Healthy Controls (pg/ml) | p | |
| --- | --- | --- | --- | --- | --- |
| CXCL11 | 39.11  (23.82~54.82) | 27.46  (19.14~36.68) | 18.65  (16.08~22.74) | NSV-SV | - |
|  |  |  |  | NSV-C | <0.001 |
|  |  |  |  | SV-C | <0.001 |
| IFNγ | 36.3  (21.41~62.04) | 33.64  (23.06~43.89) | 12.06  (8.13~16.45) | NSV-SV | - |
|  |  |  |  | NSV-C | <0.001 |
|  |  |  |  | SV-C | <0.001 |
| IL15 | 2.62  (2.13~3.65) | 3.07  (2.24~3.96) | 1.79  (1.42~2.35) | NSV-SV | - |
|  |  |  |  | NSV-C | <0.001 |
|  |  |  |  | SV-C | <0.001 |
| IL6 | 2.57  (1.98~4.08) | 2.64  (1.83~3.78) | 0.54  (0.29~0.80) | NSV-SV | - |
|  |  |  |  | NSV-C | <0.001 |
|  |  |  |  | SV-C | <0.001 |
| CXCL10 | 979.24  (787.71~1346.01) | 818.08  (537.94~1182.18) | 339.21  (218.75~502.75) | NSV-SV | - |
|  |  |  |  | NSV-C | <0.001 |
|  |  |  |  | SV-C | <0.001 |
| CXCL9 | 163.61  (126.75~222.6) | 141.52  (110.99~189.13) | 74.5  (40.75~129.25) | NSV-SV | - |
|  |  |  |  | NSV-C | <0.001 |
|  |  |  |  | SV-C | <0.001 |

**Supplementary Table1. plasma cytokine levels of different type vitiligo patients and healthy controls**

*The data in the table represent the median (interquartile range, IQR).
