# Supplementary material for: Candidate approaches for predicting vitiligo recurrence: an effective model and biomarkers
Source: Front Immunol. 2025 Feb 6;16:1468665. doi: 10.3389/fimmu.2025.1468665 (PMC11839629; doi:10.3389/fimmu.2025.1468665)
Supplement: Supplementary file 1 [file Presentation1.zip › FIG2 statistical data.docx]

| factors | Active Vitiligo (pg/ml) | Stable Vitiligo (pg/ml) | Healthy Controls (pg/ml) | p | |
| --- | --- | --- | --- | --- | --- |
| CXCL11 | 33.01  (24.6~52.63) | 30.26  (17.87~50.89) | 18.65  (16.08~22.74) | Active-Stable | - |
|  |  |  |  | Active-Controls | <0.001 |
|  |  |  |  | Stable - Controls | <0.001 |
| IFNγ | 42.88  (28.33~55.2) | 32.26  (21.3~55.16) | 12.06  (8.13~16.45) | Active-Stable | - |
|  |  |  |  | Active-Controls | <0.001 |
|  |  |  |  | Stable - Controls | <0.001 |
| IL15 | 2.69  (2.21~3.62) | 2.84  (2.13~3.79) | 1.79  (1.42~2.35) | Active-Stable | - |
|  |  |  |  | Active-Controls | <0.001 |
|  |  |  |  | Stable - Controls | <0.001 |
| IL6 | 2.77  (1.98~4.51) | 2.51  (1.85~3.82) | 0.54  (0.29~0.80) | Active-Stable | - |
|  |  |  |  | Active-Controls | <0.001 |
|  |  |  |  | Stable - Controls | <0.001 |
| CXCL10 | 960.2  (739.94~1284.61) | 948.58  (555.72~1230.58) | 339.21  (218.75~502.75) | Active-Stable | - |
|  |  |  |  | Active-Controls | <0.001 |
|  |  |  |  | Stable - Controls | <0.001 |
| CXCL9 | 176.14  (141.52~228.99) | 137.78  (111.63~195.71) | 74.50  (40.75~129.25) | Active-Stable | <0.05 |
|  |  |  |  | Active-Controls | <0.001 |
|  |  |  |  | Stable - Controls | <0.001 |

**Supplementary Table 2. plasma cytokine levels of Active Vitiligo, Stable Vitiligo patients and healthy controls**

*The data in the table represent the median (interquartile range, IQR).
