# Supplementary figures and images for: Candidate approaches for predicting vitiligo recurrence: an effective model and biomarkers
Source: Front Immunol. 2025 Feb 6;16:1468665. doi: 10.3389/fimmu.2025.1468665 (PMC11839629; doi:10.3389/fimmu.2025.1468665)

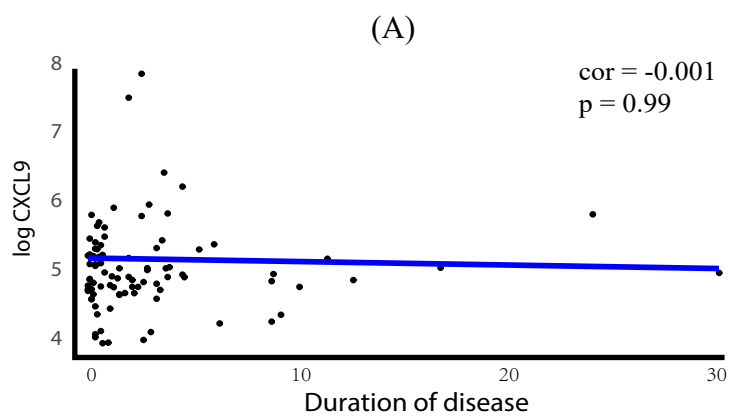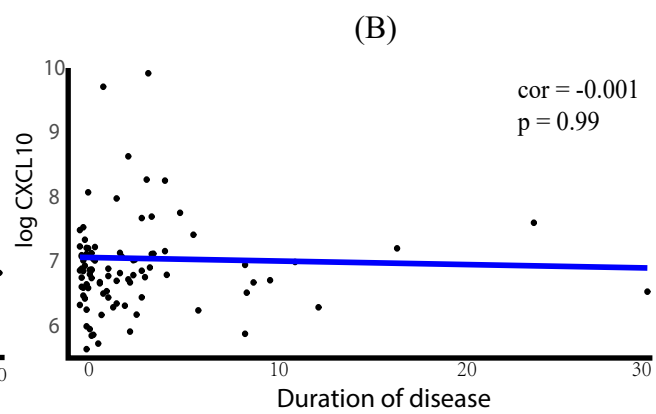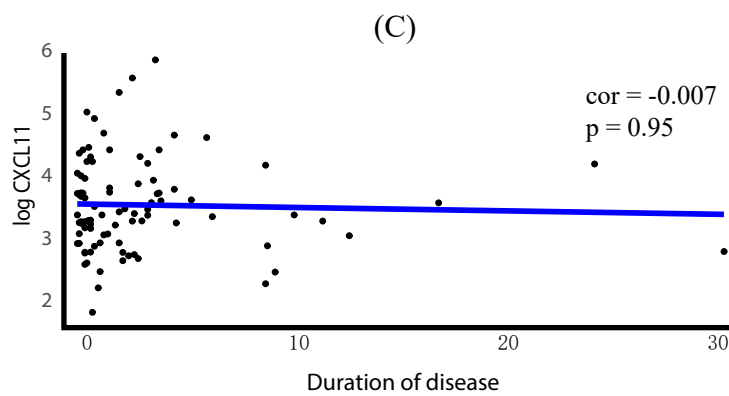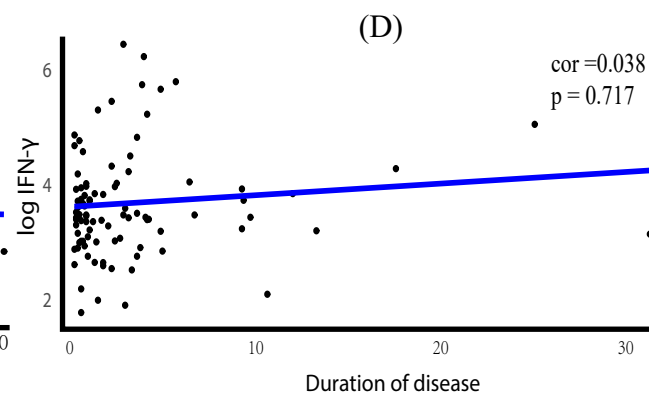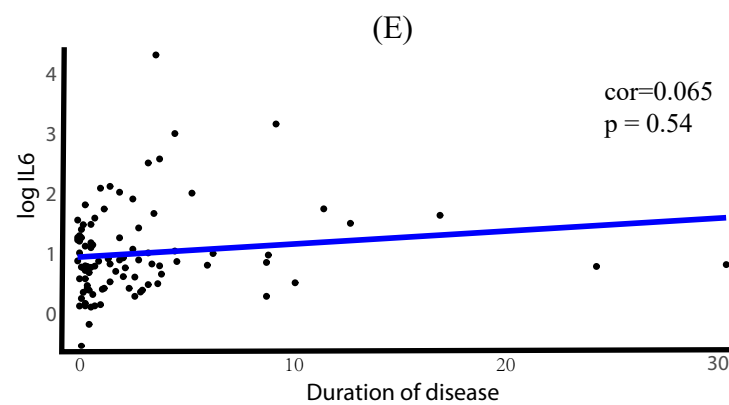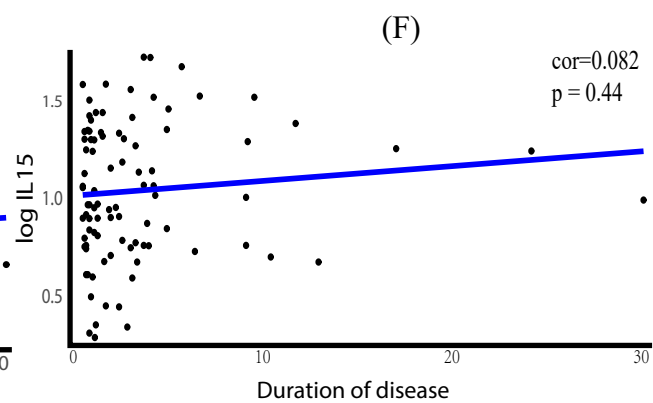

Supplement: Supplementary file 1 [file Presentation1.zip › Supplementary Fig1.pdf]
